# Gaze Behavior Effect on Gaze Data Visualization at Different Abstraction Levels

**DOI:** 10.3390/s21144686

**Published:** 2021-07-08

**Authors:** Sangbong Yoo, Seongmin Jeong, Yun Jang

**Affiliations:** Computer Engineering and Convergence Engineering for Intelligent Drone, Sejong University, Seoul 05006, Korea; usangbong@gmail.com (S.Y.); qnqbw@naver.com (S.J.)

**Keywords:** gaze data visualization, gaze behavior, machine learning

## Abstract

Many gaze data visualization techniques intuitively show eye movement together with visual stimuli. The eye tracker records a large number of eye movements within a short period. Therefore, visualizing raw gaze data with the visual stimulus appears complicated and obscured, making it difficult to gain insight through visualization. To avoid the complication, we often employ fixation identification algorithms for more abstract visualizations. In the past, many scientists have focused on gaze data abstraction with the attention map and analyzed detail gaze movement patterns with the scanpath visualization. Abstract eye movement patterns change dramatically depending on fixation identification algorithms in the preprocessing. However, it is difficult to find out how fixation identification algorithms affect gaze movement pattern visualizations. Additionally, scientists often spend much time on adjusting parameters manually in the fixation identification algorithms. In this paper, we propose a gaze behavior-based data processing method for abstract gaze data visualization. The proposed method classifies raw gaze data using machine learning models for image classification, such as CNN, AlexNet, and LeNet. Additionally, we compare the velocity-based identification (I-VT), dispersion-based identification (I-DT), density-based fixation identification, velocity and dispersion-based (I-VDT), and machine learning based and behavior-based modelson various visualizations at each abstraction level, such as attention map, scanpath, and abstract gaze movement visualization.

## 1. Introduction

In behavioral research with eye-tracking, the fixation generally refers to the act of the eye staying at informative RoI (Regions of Interest), and the saccade is the term used to describe the rapid movements between fixations [1]. We extract fixations and saccades from eye movement data to interpret the eye movements and stops of an observer. We can use various fixation identification algorithms, including velocity-based, dispersion-based, and density-based algorithms. In particular, Velocity-Threshold Identification (I-VT), which is one of the velocity-based algorithms, is a high-performance and straightforward technique and has been employed widely in many eye-tracking studies [2,3,4]. However, since the I-VT algorithm is sensitive to the velocity value near the threshold, the gaze points can be clustered in a more or less number of fixations than one of the expected fixations. Additionally, scientists utilize dispersion-based algorithms such as Dispersion-Threshold Identification (I-DT). In a few cases [5,6], the Density-Based Spatial Clustering of Applications with Noise (DBSCAN) [7] is adapted to eye movement data.

Gaze data visualization intuitively represents eye movement data over visual stimuli. Popular gaze data visualization includes heatmap (or fixation map) [8] and scanpath visualization [9]. We can grasp the gaze data abstractly with the heatmap, but we are not able to identify the gaze movements. Since it is challenging to distinguish movement behaviors within the heatmap, many scientists are in danger of interpreting eye movements according to their desired results or their preferred theory [10]. Scanpath visualization enables to analyze eye movement flows and patterns, but scanpaths are overlaid over time, which causes visual clutters. Scientists often have difficulty in interpreting gaze data due to overlapping scanpaths [11]. In addition, in the scanpath visualization, it is difficult to reveal smooth pursuit [12], which is following the moving targets.

The velocity range [13] for smooth pursuit is similar to one for the slow saccade [14], it is difficult to set the velocity threshold to distinguish the two eye movements from fixation and saccade [15]. Therefore, algorithms using machine learning techniques [16,17,18] have been proposed to improve the performance of eye movement events classification. These machine learning algorithms classify fixation, saccade, and smooth pursuit with higher accuracy than parameter thresholding techniques. However, the annotations of the ground truth data for evaluation do not have a high agreement among the experts, and improving classification accuracy is still a challenge problem [18]. In addition, machine learning techniques classify discrete eye movement events because they train models based on annotated data but do not capture continuous gaze behavior. Human gaze behavior makes smooth pursuit eye movements to place the target at the center of the fovea (also called foveal vision) when gazing at a moving target [19,20]. Since the eye is not entirely still, the fixation includes a minute movement, tremor, around the 90 Hz, a fast micromovement, microsaccades, that tends to return to the original eye position, and a slow movement, drifts, that is away from the center of fixation [10]. Additionally, the fixation occurs within 1–2° from the foveal vision center, and this foveal area is considered the range that a human can focus on [21]. In this study, for the gaze behavior analysis, we define the fixation and smooth pursuit as stare because both fixation and smooth pursuit are actions to place the target at the center of the fovea and stare at the target, and the movement like saccade as move.

The gaze visualization changes dramatically according to the fixation identification algorithms because it abstractly visualizes the gaze data except for the raw gaze point visualizations. We generally apply a fixation identification algorithm suitable for the environment of eye-tracking research by referring to the studies comparing the performance of fixation identification algorithms [22,23,24,25]. These studies save time and effort in testing all of the various fixation identification algorithms every time we design an experiment. However, since there is no study comparing the effect of fixation identification algorithms on gaze data visualizations, we tend to spend much time on selecting various visualization techniques and fixation identification algorithms during the gaze data analysis.

In this paper, we compare the effects of gaze-parsing algorithms on gaze visualizations along with different levels of abstraction. We utilize heatmaps, abstract gaze movement visualization, and scanpath visualizations for the comparison. We employ an improved technique that emphasizes the directionality of eye movements in work proposed by Yoo et al. [6] as an abstract gaze movement visualization. Moreover, we propose a behavior-based gaze data processing to classify gaze behavior into stare and move, and transform raw gaze points into image windows. The images generated by the behavior-based gaze data processing are trained with machine learning models, such as CNN (Convolutional Neural Network) [26], AlexNet [27], and LeNet [28], and applied to classify gaze behaviors. The fixation identification algorithms for the comparison include the velocity-based algorithm I-VT, the dispersion-based algorithm I-DT, the improved density-based algorithm DBSCAN with the IQR (Interquartile Range), velocity and dispersion based algorithm I-VDT, machine learning based alogrithm REMoDNaV, and the behavior-based data processing method with machine learning models. The contributions of our study are summarized as follows:

- We compare and analyze how gaze bahavior affects gaze data visualizations at different levels of abstraction.
- We propose a behavior-based gaze data processing with machine learning models.
- We improve an abstract gaze movement visualization and gaze-parsing method by extending the visualization technique presented by Yoo et al. [6].

## 2. Related Work

The heatmap visualization is utilized to interpret the distribution of gaze data. The gaze distribution enables scientists to measure how long an observer examines areas within a visual stimulus [29,30,31]. The heatmap visualization is also applied in the various visual stimulus analysis, such as the gaze data analysis in physical 3D shapes [31] and immersive video [30]. Smith and Mital [29] investigate how the gaze distribution changes according to the viewing conditions of video stimuli such as free-view and spot-the-location, and the scene types such as static and dynamic. However, the heatmap visualization primarily focuses on showing the data density. Since the density is computed with all accumulated data, the heatmap visualization is not suitable for analyzing data accumulation patterns and eye movement patterns over time.

On the other hand, scanpath visualization is an analytical technique that reveals the eye movements with fixation and saccade [32,33,34,35]. The scanpath visualization is adopted to investigate eye movement patterns in various domains [36,37]. Burch et al. [36] analyze gaze trajectories of observers looking at traditional, orthogonal, and radial layouts diagrams. Eraslan et al. [37] analyze the scanpaths to improve the usability of web pages. The scanpath visualization also shows various information in gaze data with node size, link thickness, and colors of nodes and links in addition to the movement feature [38,39,40]. Fuhl et al. [38] visualize similar gaze movement patterns between observers with color-coded links that indicate the gaze directions. Andrienko et al. [39] encode the number of gaze visits to AoIs (Area of Interests) with the line thickness, and Kurzhals and Weiskopf [40] analyze the attention of an observer by representing the fixation time as the size of the fixation node.

There are a few studies for abstracted gaze motion visualizations [6,41]. Peysakhovich and Hunter [41] extract the fixations and saccades and perform saccade bundling in the saccade direction. They propose a color-encoded visualization of the saccade length, time stamp, and saccade direction into a bundled saccade link. Yoo et al. [6] represent heatmaps by dividing eye movement data into fixation layers. They propose a gaze movement visualization applying the smudge effect on each layered heatmap. The difference between these two abstraction gaze movement visualizations is the use of temporal information. The visualization proposed by Peysakhovich and Hunter [41] focuses on the representation of the saccade directions rather than the distribution of the data over time, while the visualization proposed by Yoo et al. [6] focuses on showing eye movements with the gaze distribution over time.

Since the techniques [42,43,44,45] have been improved recently to lower the calibration errors in various conditions such as real-time, calibration-free, and head-free environments, eye tracking has been utilized in various studies. In general, researchers mainly apply statistical analysis techniques to understand eye movements [46,47,48]. Besides, gaze data visualization is also employed to obtain additional insight along with statistical analysis techniques [49,50]. Both statistical and visualization techniques are utilized for analysis mainly after extracting eye movement events from raw gaze points. Fixation identification algorithms as the event extraction techniques include velocity-based, dispersion-based, and density-based algorithms [1,5,22,23,25]. The most representative algorithm of the velocity-based fixation identification algorithm is I-VT [1] that produces fixations with one parameter, the velocity threshold. Many scientists use the I-VT due to its simplicity and relatively good performance. In particular, companies that design eye-tracking devices like Tobii (Tobii AB, https://www.tobii.com/, accessed on 8 July 2021) also employ the I-VT; therefore, we can easily utilize this algorithm during the use of commercial eye-tracking devices. However, the I-VT has a problem of blips that are sensitive to velocity near the threshold and create undesirable fixations [1]. Therefore, scientists apply I-HMM (Hidden Markov model Identification) [1,25] for more robust identification than the I-VT. Tobii also provides higher performance fixation identification algorithms with additional features such as noise filtering along with the I-VT [2]. I-DT is a dispersion-based fixation identification algorithm that uses two parameters, dispersion and duration. Llanes-Jurado et al. [51] propose a new algorithm with I-DT for the fixation identification in immersive virtual environments. Since they reflect the VR-centered paradigm, apply the 3D head position and 3D points of gaze rays that intersect virtual objects into the algorithm. Density-based fixation identification algorithms are not commonly used but there are a few studies [5,6,52]. Yu et al. [5] propose I-STTraDBSCAN, which modifies Eps and MinTime as parameters for the gaze fixation identification. Yoo et al. [6] apply DBSCAN, which is a time-weighted eye movement data, with the interquartile range (IQR) as a fixation identification algorithm in the gaze visualization. Liu et al. [52] present an outlier-aware fixation identification algorithm that extends the FID (fixation-inner-density) filter. Machine learning is also applied for fixation identification [16,53,54]. Akshay et al. [53] shows that the random forest and decision tree algorithms have the highest accuracy for the fixation and saccade classification among K-Means, KNN, SVM, Decision Tree, and Random Forest using the fixation dataset labeled with I-DT. The eye movements classification models proposed by Zemblys et al. [54] and Startsev et al. [16] do not require parameters and thresholds. Zemblys et al. [54] introduce *gazeNet*, a framework for creating event detectors using deep neural network, which classifies fixation, saccade, and PSO (post-saccadic oscillations). Startsev et al. [16] present a deep learning system for fixation, saccade, and smooth pursuit classification. Their system utilizes 1D CNN with BLSTM to classify eye movement events. In addition to these mentioned in this section, more gaze-parsing algorithms can be designed by selecting various algorithms depending on the applications [55,56,57].

## 3. Method

In this paper, we aim to qualitatively compare the effects of gaze-parsing algorithms on gaze data visualizations at different levels of abstraction. We first compare scanpaths and the number of fixations according to the parameter settings of fixation identification algorithms (see Section 4.1). In the next step, we propose a behavior-based gaze data processing model for the intermediate abstraction level of gaze data visualization, which is in between heatmap visualization and scanpath visualization (see Section 4.2). Lastly, we qualitatively compare the effect of the gaze-parsing algorithm on gaze data visualizations at different levels of abstraction (see Section 5). In this section, we introduce the heatmap, scanpath, and mid-level abstraction gaze data visualization utilized for comparison. We also describe the eye-gaze tracker employed to collect eye movement data, visual stimulus and task, and datasets used to train and test the model.

### 3.1. Different Abstraction Level Gaze Data Visualizations

**Figure 1:**
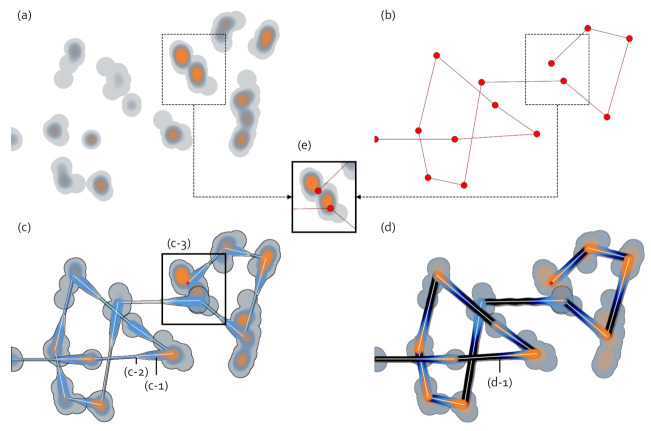
Gaze visualizations. (**a**,**b**) The heatmap and scanpath visualization. (**c**) Our abstract gaze movement visualization. (**d**) A gaze data visualization proposed by Yoo et al. [6]. (**e**) presents the visualization of combining the heatmap and scanpath visualizations. (**c-1**) and (**d-1**) shows an example of eye movements and (**c-2**) indicates the tapering on the path for the direction. (**c-3**) includes complicated eye movement patterns that correspond to (**e**).

In this section, we briefly introduce the gaze data visualizations used in our study. Figure 1 shows examples of the gaze visualizations. Figure 1a,b are the heatmap and scanpath visualizations that we typically find in many gaze studies. In our study, (a) indicates the visualization at the high abstraction level, and (b) denotes the visualization at the low abstraction level. (c) and (d) present the abstract gaze movement visualizations. (c) is a modified visualization from the visualization in (d) proposed by Yoo et al. [6]. We utilize the visualization in (c) as a visualization at the mid-level abstraction. We have enhanced the visualization in (d) to produce more intuitive visualization for data directionality, as seen in (c). The eye movements between the heatmaps are marked in (c-1) and (d-1). In (d), the eye movement is represented with a constant thickness, as shown in (d-1). Additionally, as the smudge effect is applied, the color of the eye movement is turned to black and emphasized unnecessarily. On the other hand, we visualize the eye movement as presented in (c-1), with the smudge effect slightly pulled from the heatmap, so that the eye movements are visualized separately in the overlapping areas. We show the directionality compactly by tapering and reducing the thickness of the eye movement, as shown in (c-2). The color of the eye movement stretches from the color of the source to the color of the destination. Thus, this color selection eliminates unnecessary stresses, allowing us to focus more on eye movement directions.

Figure 1e is a combination of the heatmap and scanpath visualization at the same location as (c-3), and only the scanpath is drawn on the heatmap that represents the density of the entire data regardless of time. Contrary to this, in (c-3), the heatmap layers are divided over time.

### 3.2. Eye-Gaze Tracker

We compare fixation identification algorithms in Section 4.1 and collect eye movement data to create a behavior-based gaze data processing model in Section 4.2. Additionally, in Section 5, we compare the effects of gaze-parsing algorithms on gaze data visualizations. We collected the gaze movement data using the 40 Hz screen-based eye tracker, Tobii Pro X2-30 (Tobii Pro X2-30, https://www.tobiipro.com/product-listing/tobii-pro-x2-30/, accessed on 8 July 2021). The Tobii Pro X2-30 has a lower sampling frequency than the more professional Tobii Pro eye tracker (Tobii Pro Fusion, https://www.tobiipro.com/product-listing/fusion/, accessed on 8 July 2021) or EyeLink 1000 (EyeLink 1000 Plus, https://www.sr-research.com/eyelink-1000-plus/, accessed on 8 July 2021). However, Tobii Pro X2-30 is being used in various studies for analyzing human gaze behavior [58,59,60,61,62,63]. Additionally, Tobii Pro SDK (Tobii Pro SDK, http://developer.tobiipro.com/index.html, accessed on 8 July 2021) and eye tracker manager software (Tobii Pro Eye Tracker Manager, http://developer.tobiipro.com/eyetrackermanager.html, accessed on 8 July 2021) for professional eye-tracking research are provided. The eye tracker collects the eye movement data of the observer more similarly to reality as the sampling rate increases. However, the higher the sampling rate, the more expensive the eye tracker. Table 1 shows the F1 scores of event classification for eye movement data collected using 500-Hz and 40-Hz eye trackers. The data for 500-Hz included the eye movement data measured while watching BergoDalbana.avi, BiljardKlipp.avi, TrafikEhuset.avi, and triple_jump.avi videos in the Lund2013 dataset (Available for download at https://github.com/richardandersson/EyeMovementDetectorEvaluation, accessed on 8 July 2021) distributed by Larsson et al. [15]. We collected the data for 40-Hz with Tobii Pro X2-30 for the algorithm comparisons in Section 5. I-VDT [64] and REMoDNaV [65] are algorithms for smooth pursuit classification. The I-VDT takes velocity and dispersion threshold as parameters. The REMoDNaV is a parameter-free algorithm based on machine learning. When comparing the F1 scores for the two datasets measured at 500 Hz and 40 Hz in Table 1, there is a slight difference with up to 0.08 for smooth pursuit, up to 0.09 for fixation, and up to 0.02 for saccade between these two datasets. Therefore, we believed that the measurement sampling rate (40-Hz) of the Tobii Pro X2-30 used in this study would not significantly affect the results. Note that we calibrated the experiment environment using the eye tracker manager software provided by Tobii. In addition, to reduce noise caused by eye blink, raw gaze data points not detected in both left and right eyes in Tobii pro SDK were treated as the outliers.

### 3.3. Recruitment of Observers

We recruited 10 observers for data collection. Two undergraduate and three graduate students majored in computer science. The remaining five were recruited regardless of their majors. The observers were all adults who were not wearing glasses, and there were six observers in their 20s and four observers in their 30s. Since the number of fixations and gaze data visualizations according to the parameter setting of the fixation identification algorithms did not change by the observers, and the comparison among the observers was not the purpose of the study, we randomly selected data from 1 out of 10 observers.

### 3.4. Visual Stimulus and Task

**Figure 2:**
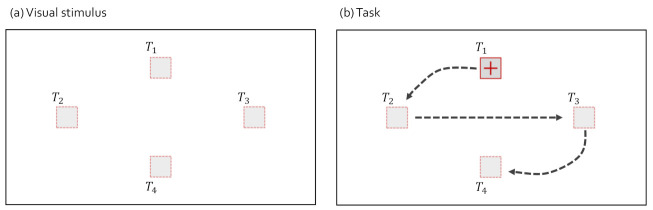
Visual stimulus and the task to collect gaze data for comparisons. We have used Tobii Pro X2-30.

For the analysis in Section 4.1, the visual stimulus and task as shown in Figure 2 were used for the comparison of gaze-parsing algorithms. Note that we did not apply a natural visual stimulus in this paper to prevent the observer’s gaze from moving to an unintended place. The observers stared at the visual stimulus presented in Figure 2a and followed the box target moving along the path presented in Figure 2b. The box target moved in order, $T_{1}$, $T_{2}$, $T_{3}$, and $T_{4}$. We controlled the experiment time within 20 s. We have designed various periods of each box target staying at each target stimulus location. We collected data with the tasks having various transition times of the target moving from $T_{1}$ to $T_{4}$ and selected the task that best showed the problems of I-VT, I-DT, DBSCAN with IQR, and I-VDT in Section 4.1. We created the tasks by combining the transition times of the target. We designed 27 tasks with transition time combinations of 0.1, 0.2, and 0.5 s and four tasks with combinations of 1 and 2 s. Note that the distance between $T_{1}$ and $T_{2}$, and $T_{3}$ and $T_{4}$ is 15 degrees, and the distance between $T_{2}$ and $T_{3}$ is 27.5 degrees. The time the target stayed in $T_{1}$, $T_{2}$, $T_{3}$, and $T_{4}$ was fixed as 3 s. The transition time of the moving target used in the analysis of each fixation identification algorithm is described in each subsection of Section 4.1.

For the analysis in Section 5, eye movement data were collected using 6-point stimuli. Data were gathered through four task combinations with moving patterns and whether the moving target stopped at six points. The moving target speed was fixed at 30 deg/s. There were two eye-moving patterns, including repeating up and down and running in Z-shape. There were two stop patterns of moving target, including one case that the moving target stayed at all six points for 3 s and another case that the moving target passed without pausing. The moving patterns and stop patterns of the moving target utilized in the visualization comparison according to the gaze data visualization are presented in each subsection of Section 5.

### 3.5. Ground-Truth Data

We examined the fixation classification performance of CNN, AlexNet, and LeNet machine learning models with the training data generated by our proposed gaze behavior-based data processing in Section 4.2. We utilized the eye movement data as training and validation datasets. As the test dataset, we used the Lund2013 dataset [15] that included labeled eye movement events as fixation, saccade, smooth pursuit, PSO (post-saccadic oscillations), blinks, and unknown. We investigated the models by classifying fixation and smooth pursuit into the stare label and saccade and PSO into the move label. Note that in our test, the smooth pursuit was set to stare because it was an action staring at a moving target rather than a moving action, and PSO to move because PSO occurred only after saccade [54].

## 4. Gaze-Parsing Algorithms

In this section, we identify problems with fixation algorithms that require parameter settings and introduce a behavior-based gaze data processing method with an image-based machine learning classification algorithm.

### 4.1. Problems with Manual Parameter Settings

The fixation identification algorithms used in most eye-tracking studies require manual parameter settings. In particular, I-VT, I-DT, and DBCAN with IQR have a velocity threshold (see [1,2] for equations), dispersion and duration thresholds (see [1] for equations), eps and minPoint as parameters, respectively. Many scientists utilize fixation identification algorithms in eye-tracking studies, but it is difficult to determine the ideal fixation parameters because they must adjust parameters manually, relying on their experiences. To analyze fixation identification algorithms according to parameter settings of the fixation identification algorithms, we collected gaze data in a tightly controlled environment as mentioned in Section 3.

#### 4.1.1. I-VT Fixation Identification Algorithm

**Figure 3:**
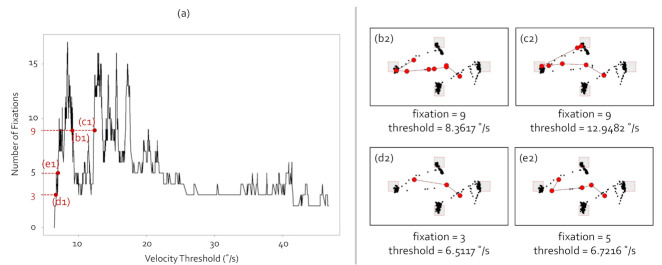
I-VT fixation identification. (**a**) shows the number of fixations according to the velocity threshold. The visualizations of (**b2**), (**c2**), (**d2**), and (**e2**) include the raw gaze points and scanpaths at (**b1**), (**c1**), (**d1**), and (**e1**) in (**a**), respectively. (**b2**) to (**e2**) show four cases of fixation identifications. The black dots denote the raw gaze points, and the red dots indicate the fixations. The red links are the saccades.

Figure 3 shows the fixations of the gaze data with the I-VT algorithm. In this analysis, we utilized gaze data staring at the visual stimulus where the box target stayed on all targets ($T_{1}$ to $T_{4}$) for 3 s and equally moved for 0.5 s between targets. Figure 3a presents the number of fixations according to the velocity threshold. Subfigures (b1), (c1), (d1), and (e1) show the scanpaths when the number of fixations extracted in order was 9, 9, 3, and 5, respectively. As seen in Figure 3a, the number of fixations changed irregularly as the velocity threshold increased. The visualizations of (b2) to (e2) show the raw gaze points and scanpaths at (b1) to (d1) in (a), respectively. Subfigures (b2) and (c2) on the right images show different scanpath shapes even though the number of fixations was the same. Subfigures (d2) and (e2) on the right images have different fixation numbers and scanpath shapes, although the velocity thresholds were almost same. Subfigures (d1) and (e1) had similar velocity thresholds. The velocity thresholds of (d1) and (e1) were 6.5117 and 6.7216, respectively, as seen in (d2) and (e2). However, the numbers of fixations were different. The I-VT fixation identification algorithm was greatly affected by minute changes in velocity threshold due to its simple structure using only one parameter. The blips problem, that one fixation was identified as multiple fixations since I-VT reacted sensitively at the velocity threshold boundary, also occurred for the same reason.

#### 4.1.2. I-DT Fixation Identification Algorithm

**Figure 4:**
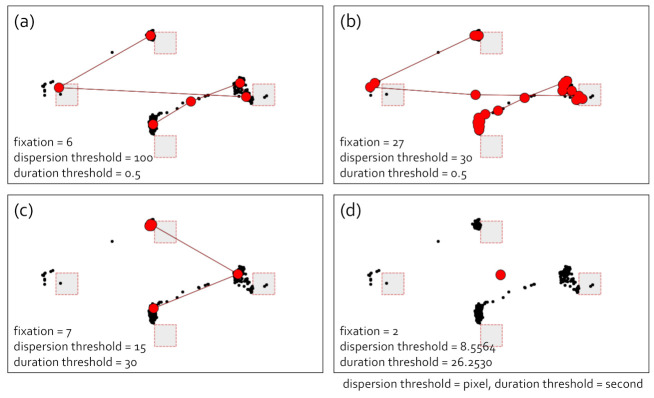
I-DT fixation identification. (**a**–**d**) The different cases of fixation identifications with the I-DT algorithm. The parameter settings are displayed in each image. Similar to Figure 3, the black dots denote the raw gaze points, and the red dots indicate the fixations. The red links are the saccades.

Figure 4 presents the fixations of the gaze data using the I-DT algorithm. The gaze data we used in this analysis were obtained as the box target stayed for 3 s on the targets, $T_{1}$, $T_{3}$, and $T_{4}$, and 0.2 s on $T_{2}$. Additionally, it took 0.1 s for the box target to move from $T_{1}$ to $T_{2}$ and from $T_{2}$ to $T_{3}$, whereas it took 0.5 s to move the box target from $T_{3}$ to $T_{4}$. As shown in Figure 4a,b, there are two similar scanpaths with a different number of fixations. However, this was the case when we fixed one parameter and adjusted only the other parameter. Additionally, even if we could accidentally obtain the ideal value of a parameter and manipulate only the other, we still were not sure whether we discovered the ideal number of fixations. Subfigures (c) and (d) in Figure 4 show the cases where fixations were not appropriately extracted according to the parameter setting because two parameters in the I-DT algorithm are closely associated. The scanpath in (c) did not contain a fixation at $T_{2}$, and the scanpath in (d) did not include most fixations.

#### 4.1.3. DBSCAN with IQR

**Figure 5:**
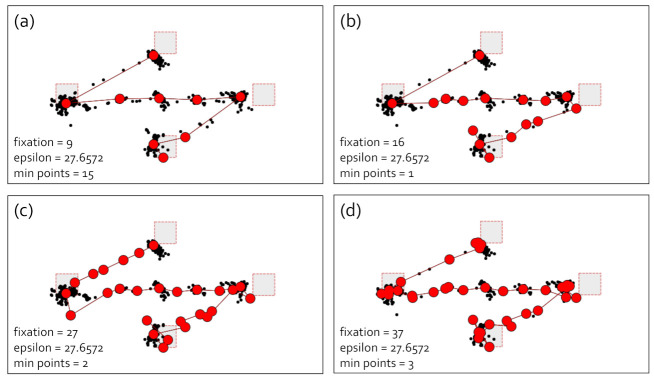
DBSCAN with IQR fixation identification. (**a**–**d**) The different cases of fixation identifications. The minPoint parameter in each case is presented in each image. The epsilon (eps) is automatically set based on Q3, the third quartile (75%), from the center of gaze distributions [6]. Similar to Figure 3, the black dots denote the raw gaze points, and the red dots indicate the fixations. The red links are the saccades.

Figure 5 shows the fixations of the gaze data using DBSCAN with IQR. In this analysis, the box target stayed for 3 s at every target in the stimulus. The box target moved for 1 s, 2 s, and 1 s from $T_{1}$ to $T_{2}$, $T_{2}$ to $T_{3}$, and $T_{3}$ to $T_{4}$, respectively. DBSCAN with IQR automatically set the eps value optimized for the gaze data in the DBSCAN algorithm (see [6]). Thus, as seen in Figure 5a–d, all scanpaths looked similar. However, DBSCAN with IQR had a big difference in the number of fixations depending on the other parameter, minPoint.

#### 4.1.4. I-VDT Smooth Pursuit Identification Algorithm

**Figure 6:**
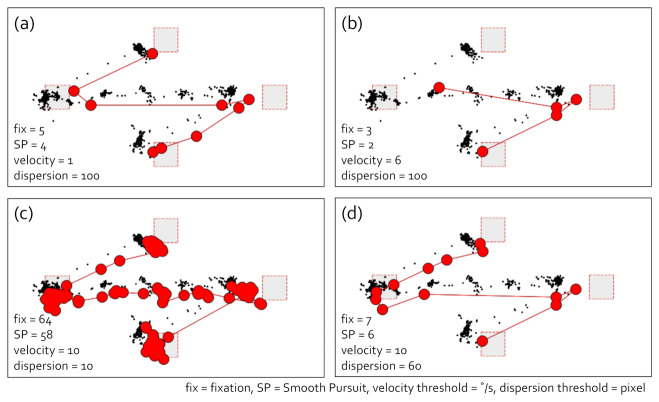
I-VDT smooth pursuit identification. (**a**–**d**) The different cases of eye movement event identifications. The velocity and dispersion threshold parameters in each case are presented in each image. The black dots denote the raw gaze points, and the red dots indicate the fixations and smooth pursuits. The red links are the saccades.

Figure 6 shows the identification of eye movement events using I-VDT. In this analysis, the box target stayed for 3 s at every target in the stimulus. The box target moved for 1 s, 2 s, and 1 s from $T_{1}$ to $T_{2}$, $T_{2}$ to $T_{3}$, and $T_{3}$ to $T_{4}$, respectively. Since the velocity range of smooth pursuit overlapped with the saccade, the parameter setting was challenging. Subfigures (a) and (b) show that the eye movement event identification varied differently depending on the velocity threshold for the same dispersion threshold. The smooth pursuit was sometimes mixed with fixation. Subfigure (c) reveals that too many fixations and smooth pursuits were classified because the dispersion threshold was too low compared to (d).

### 4.2. Behavior-Based Gaze Data Processing

As presented in the previous section, the fixation identification algorithms with parameter settings such as I-VT, I-DT, and DBSCAN with IQR extract the different number of fixations and the different scanpath shapes depending on the parameter settings. Most scientists apply these algorithms, but spend much time and effort on determining appropriate parameters. Since the velocity range of smooth pursuit overlaps the velocity range of saccade, it is difficult to set the velocity threshold for the smooth pursuit detection [13,15]. To resolve the problem, we split eye movement data into stare and move based on gaze behavior rather than events. Since the fixation and smooth pursuit reflected the gaze behavior of the observer who wanted to gaze at the target, it could be identified as stare, and other movements could be recognized as move. Note that this definition did not simply mean combining fixation and smooth pursuit into one label. Existing machine learning techniques for smooth pursuit detection classify discrete eye movement events. However, continuous gaze behavior is not recognized because the classification is achieved through learning annotated events rather than gaze behavior. Therefore, we propose a gaze-parsing technique to identify continuous gaze behavior by learning gaze behavior in this section.

**Figure 7:**
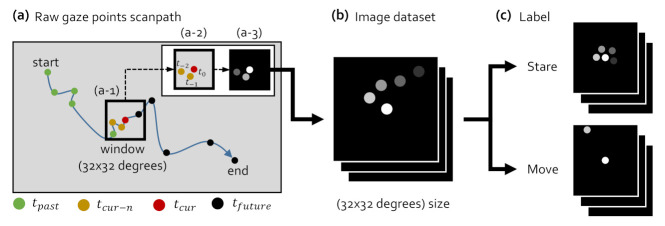
Gaze bahavior-based data processing for training with image classification machine learning algorithm. The image dataset is generated in the form of a 32 × 32 degrees window with a black background.

In this study, we introduce a behavior-based gaze data processing method using the image classification machine learning algorithms to reduce the effort and time spent on setting parameters manually and to include smooth pursuit eye movements on fixation aggregation. Figure 7 shows the bahavior-based gaze data process of creating labeled datasets from raw gaze points for the training in the image classification machine learning models. As seen in (a), we generated a virtual window with the size of a human visual angle, crop gaze points within the virtual window according to eye movement, and save cropped gaze points as an image. We implemented data visualization, cropping, and image storage with Python OpenCV library (Python OpenCV library, https://opencv.org/, accessed on 8 July 2021). Figure 7a illustrates how we generated image datasets in (b) with a 32 × 32 degrees window. Note that we determined the window size as 32 × 32 degrees since the smooth pursuit [10] moved at a speed of 10–30 degrees per second and the visual angle is 1–2 degrees [21] that humans could focus. The window moved from the start point to the end point of the raw gaze data in time order. The window was located at the center of the current gaze point ($t_{cur}$), as presented in Figure 7(a-1). Of the gaze points within the window range, the data from the current gaze point to the past *n*th, i.e., $t_{cur}$ to $t_{cur-n}$, were taken as shown in Figure 7(a-2), where n was set to 2 in this illustration, and there were three gaze points, $t_{0}$, $t_{-1}$ and $t_{-2}$, taken within the window range. As presented in Figure 7(a-3), we made the window background black and make the gaze points in Figure 7(a-2) more transparent away from the present in time order. In this process, the image data in Figure 7b were generated. The background color of the image dataset was fixed to black to discard other visual stimuli to learn only the time-dependent behavior of the scanpath of raw gaze points. We label the images in Figure 7b as *stare* and *move* according to the gaze behaviors. The proposed behavior-based gaze data processing method generates images where gaze points were gathered as the stare class when the gaze behavior stares in one area or was a smooth pursuit moving at a speed of 10–30 degrees per second. Saccades were formed as the move class, where there was a gap between gaze points or gaze points were not gathered. As illustrated in Figure 7c, the labeled data were divided into the training datasets and validation datasets. We created a total of 8000 training datasets and 8000 validation datasets through the process in Figure 7. Note that we used the same number of data from each class.

**Figure 8:**
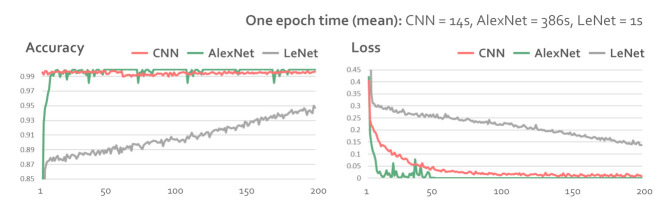
Accuracy, loss, and one epoch training mean time performance comparisons of CNN, AlexNet, and LeNet.

We compared the performance of three machine learning algorithms, including CNN, AlexNet, and LeNet, with the image data generated by the behavior-based gaze data processing method. We used the CNN model consisting of three convolutional layers, one max-pooling layer, and two fully connected layers. The ReLU (Rectified Linear Unit) activation function was used in the convolutional layers and the fully connected layer. AlexNet consisted of five convolutional layers, three max-pooling layers, and three fully connected layers. The activation function was ReLU. LeNet consisted of two convolutional layers, two max-pooling layers, and three fully connected layers. The sigmoid was applied as the activation function. The input was the image dataset we created, and the output was the classification, stare and move. Figure 8 shows the performance of the bahavior-based gaze data processing method using CNN, AlexNet, and LeNet. We calculated the accuracy and loss to compare the performance of each model. The accuracy denotes the classification accuracy defined as $accuracy=\frac{\left(TP+FP\right)}{\left(TP+FP+FN+TN\right)}$. *TP* is True Positive, *FP* is False Positive, *FN* is False Negative, and *TN* is True Negative. The loss implies the difference between the data distribution for learning and the data distribution predicted by the model. The loss is defined as H(P,Q)=−ΣP(x)logQ(x), where *H* is the loss and *P* is the distribution of the data for training. *Q* is the data distribution predicted by the model to approximate *P*, and *x* is the observation for the correct answer label. We compared the loss and accuracy of CNN, AlexNet [27], and LeNet [28] in Figure 8. AlexNet had the highest accuracy with the smallest loss, whereas LeNet had the lowest performance for both accuracy and loss. On average, it took 14 s for CNN, 386 s for AlexNet, and 1 s for LeNet per epoch during the training. The hardware configuration used for training and classification of all models was Intel i7-3770 cpu @3.4 G Hz, eight cores, NVIDIA GeForce GTX 660 Ti 4 GB memory, and 16 GB RAM.

## 5. Comparison of Gaze-Parsing Algorithms and Gaze Data Visualizations

In this section, we compare the effects of gaze-parsing algorithms on the abstract levels of gaze data visualization. We compare the heatmap, scanpath, and abstract gaze movement visualization using I-VT, I-DT, DBSCAN with IQR, and the behavior-based gaze data processing method using AlexNet. We manually set the parameters of the fixation identification algorithms by determining the optimal results based on the shape of the scanpath compared to the conditions of collecting the gaze data.

### 5.1. Heatmap Visualization

**Figure 9:**
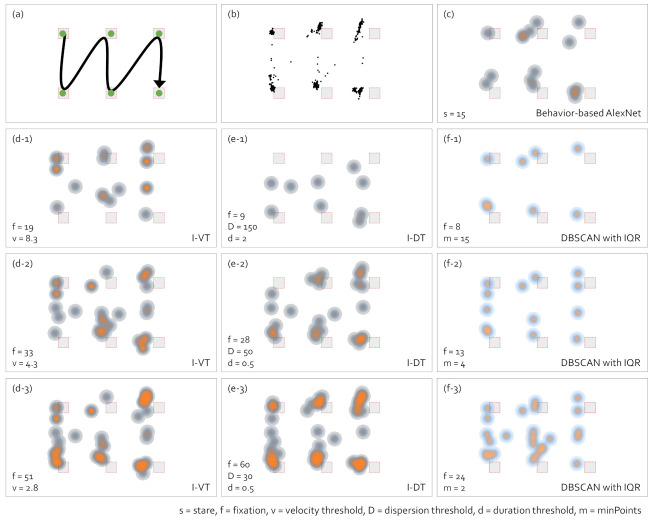
Comparison of heatmaps with different gaze-parsing algorithms. (**a**) A visual stimulus, including the eye movement task of an observer. The observer paused for 3 s at the point where the green dots are drawn during the eye movement. (**b**) The raw gaze points. (**c**) The heatmap with the behavior-based gaze data processing method using AlexNet. The heatmap with I-VT in (**d-1**–**d-3**), I-DT in (**e-1**–**e-3**), and DBSCAN with IQR in (**f-1**–**f-3**) are presented.

We chose the heatmap as a gaze visualization representing a high level of abstraction. In general, heatmaps were represented by counting raw gaze points or counting fixation points. Since we could not examine the changes according to the fixation identification algorithms with the number of raw gaze points, we represented the heatmap by counting the fixation points in this study. Figure 9 shows the changes in the heatmap according to the gaze-parsing algorithms.

In the data collection, the observer saw the visual stimulus, as presented in Figure 9a. The observer paused for three seconds at the points where the green dots are drawn while moving his eyes. Subfigure (b) shows the raw gaze points. Subfigure (c) is the heatmap with the behavior-based gaze data processing method using AlexNet. The heatmaps with I-VT, I-DT, and DBSCAN with IQR are presented in (d-1) to (d-3), (e-1) to (e-3), and (f-1) to (f-3), respectively. Note that the parameter values are printed on the visualizations. Most cases showed similar heatmaps. However, I-VT in (d-1), I-DT in (e-1), and DBSCAN with IQR in (f-1) did not produce a sufficient number of fixations. The behavior-based gaze data processing method using AlexNet did not produce fixations along the gaze path shown in (a) unlike (d-2, d-3), (e-2, e-3), and (f-2, f-3). DBSCAN with IQR identified the smaller number of fixations compared to ones with I-VT or I-DT by aggregating adjacent gaze points over both time and space coordinates. Figure 9(f-2,f-3) shows that DBSCAN was less preferred to represent the fixation density in the high level of the abstract visualization compared to (d-2, d-3) and (e-2, e-3) since I-VT and I-DT identification algorithms are sensitive to identify fixations. Therefore, in the high level of abstract visualization such as heatmap, it was necessary to consider the degree of aggregation within the fixation identification algorithm to reveal the detail of data distribution.

### 5.2. Scanpath Visualization

We used the scanpath visualization to represent a low level of abstraction. In general, the scanpath visualization is applied to analyze eye movement patterns. We compared the fixations of gaze data with an eye movement, such as searching for information and following the moving target.

**Figure 10:**
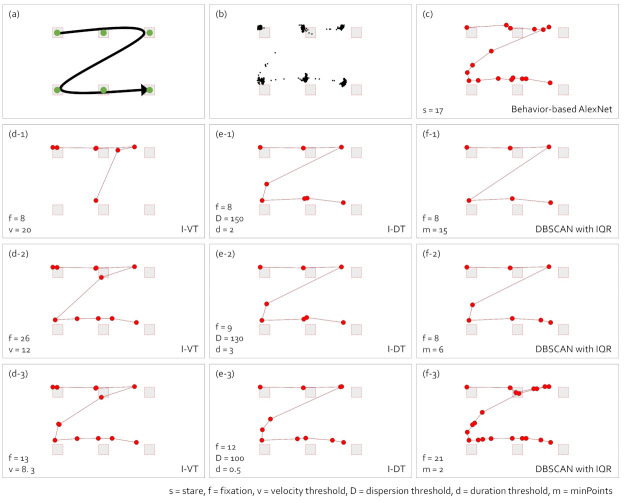
Comparison of scanpath visualizations with the eye movement according to the gaze-parsing algorithms. (**a**) The visual stimulus with the eye movement task of an observer. The observer pauses for 3 s at the points where the green dots are drawn during the eye movement. (**b**) The raw gaze points. (**c**) The scanpath visualization with the behavior-based gaze data processing method using AlexNet. The scanpath visualizations with I-VT in (**d-1**–**d-3**), I-DT in (**e-1**–**e-3**), and DBSCAN with IQR in (**f-1**–**f-3**) are presented.

Figure 10 presents scanpath visualizations by the fixation identification algorithms. The observer sees the visual stimulus in Figure 10a. The observer paused for 3 s at the points where the green dots are located. Subfigure (b) shows the raw gaze points. Subfigure (c) presents the scanpath visualization with the behavior-based gaze data processing method using AlexNet. The scanpath visualizations with I-VT, I-DT, and DBSCAN with IQR are presented in (d-1) to (d-3), (e-1) to (e-3), and (f-1) to (f-3), respectively. Note that the parameter values are printed on the visualizations. In the fast eye movement similar to searching behavior, Figure 10 shows all similar scanpaths except for (d-1), and the scanpath in (c) was similar to the ones in (d-3), (e-3), and (f-3). However, the number of fixations varied greatly depending on the parameter values.

**Figure 11:**
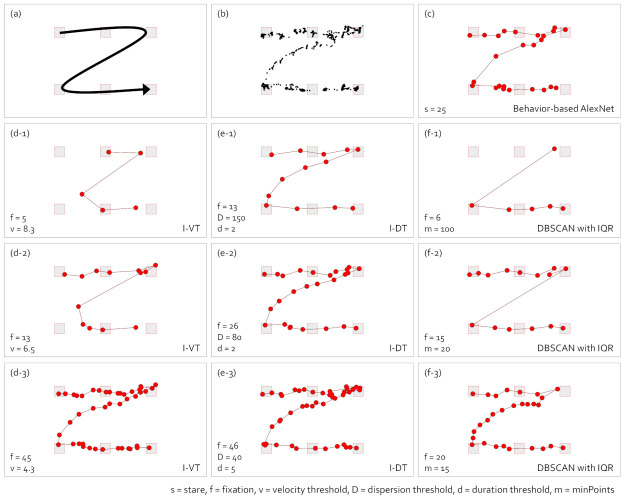
Comparison of scanpath visualizations with the eye movement while reading according to the gaze-parsing algorithms. (**a**) The visual stimulus with the eye movement task of an observer. The observer slowly moves the eye to the end without pausing the eye movement. (**b**) The raw gaze points. (**c**) The scanpath visualization with the behavior-based gaze data processing method using AlexNet. The scanpath visualizations with I-VT in (**d-1**–**d-3**), I-DT in (**e-1**-**e-3**), and DBSCAN with IQR in (**f-1**–**f-3**) are presented.

Figure 11 compares the effect of the gaze-parsing algorithms on the scanpath visualization in the smooth pursuit eye movement such as following the moving target. When the observer’s gaze followed the moving target, more scanpath visualizations lost the expected shape, as seen in (d-1), (d-2), (f-1), and (f-2), than the scanpath visualization shown in Figure 10. However, the scanpaths were visualized similar to the shape of the raw gaze points in (b) with the behavior-based gaze data processing method using AlexNet in (c), I-VT in (d-3), I-DT in (e-1) to (e-3), and DBSCAN in (f-3). In the low-level techniques such as scanpath visualization, it is essential to explore detailed eye movement patterns. As shown in Figure 11, in the searching movement, even though the number of fixations increased, the level of abstraction remained as the fixations were added only in the area where the eyes paused. On the other hand, as shown in Figure 11, the smooth pursuit eye movement showed a lower abstraction level as the number of fixations increased.

### 5.3. Abstract Gaze Movement Visualization

**Figure 12:**
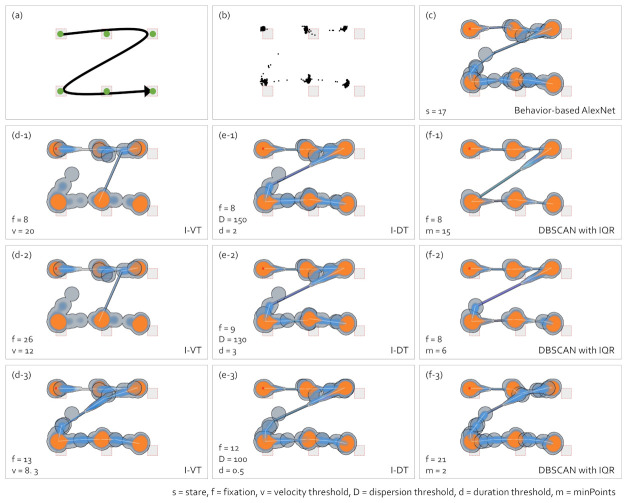
Comparison of abstract gaze movement visualizations according to the gaze-parsing algorithms. (**a**) The visual stimulus with the eye movement task of an observer. The observer pauses for 3 s at the points where the green nodes are drawn during the eye movement. (**b**) The raw gaze points. (**c**) The abstract gaze movement visualization with the behavior-based gaze data processing method using AlexNet. The abstract gaze movement visualizations with I-VT in (**d-1**–**d-3**), I-DT in (**e-1**–**e-3**), and DBSCAN with IQR in (**f-1**–**f-3**) are presented.

The abstract gaze movement visualization represents a mid-level of abstraction between the heatmap and the scanpath visualization. This visualization contains the features of both the heatmap and scanpath visualization. Figure 12 shows the abstract gaze movement visualizations according to the gaze-parsing algorithms.

As the eye movement task shown in Figure 10, the observer moved the eye along the path shown in Figure 12a and pauses for 3 s at the green dots. (b) shows the raw gaze points. In (c) to (f-3), we present the abstract gaze movement visualizations according tothe gaze-parsing algorithms shown in Figure 10.

This type of visualization became complicated when the stares were located closely, and the heatmaps were overlaid. The mid-level abstraction gaze visualization presents the ideal case at the level where the eye movement path was identified rather than the number of stare, unlike the heatmap and the scanpath visualization. The visualization in Figure 12(f-1) with DBSCAN is the ideal case for this type of visualization. In other cases, extra data post-processing might be needed after using the gaze-parsing algorithms to draw the abstract gaze movement visualizations. Since not all cases showed ideal results with DBSCAN, this visualization might require much time and effort in the post-processing. Although the parameter settings are particularly tricky in the mid-level abstraction gaze visualizations and the heatmap overlaps in most cases, the visualizations show clearly the areas where the eye paused and the eye movement directions. However, when we combined the gaze distribution with the gaze movement directions, the gaze movement path could be different, as shown in Figure 12(d-1,d-2) according to the parameter setting.

**Figure 13:**
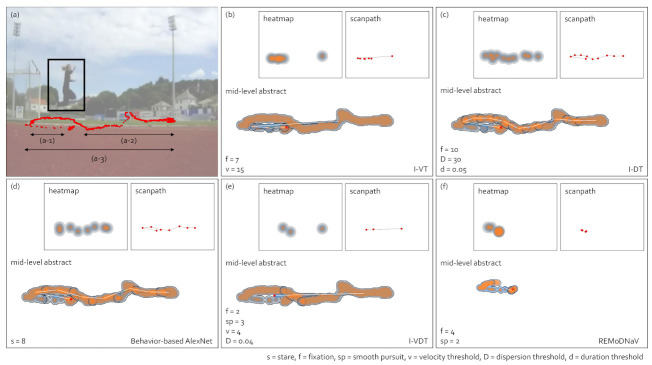
Comparison of gaze data visualizations for various gaze-parsing algorithms using Lund2013 dataset [15]. (**a**) The visual stimulus, moving target in the black box, and raw gaze points in red. (**a-1-a-3**) indicate the gaze data segments. (**b**–**d**) The results obtained using I-VT, I-DT, and our behavior-based AlexNet, respectively. (**e**) and (**f**) The results of identifying eye movement events with I-VDT and REMoDNaV, respectively. The results of each algorithm are represented in the heatmap, scanpath, and mid-level abstract gaze movement visualizations. The parameter settings and the number of detected eye movement events are disclosed in each subplot.

**Figure 14:**
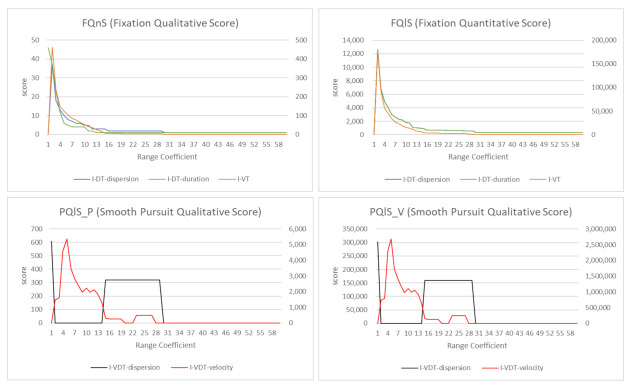
Qualitative and quantitative comparison of gaze-parsing algorithms requiring parameter setting. (**a**) The FQnS scores of I-VT and I-DT. (**b**) The FQlS scores of I-VT and I-DT. I-DT is divided into I-DT-dispersion and I-DT-duration in (**a**,**b**) because it has dispersion and duration threshold as parameters. (**c**) The PQlS_P scores of I-VDT. (**d**) The PQlS_V scores of I-VDT. In (**c**,**d**), since I-VDT has velocity and dispersion threshold as parameters, it is divided into I-VDT-velocity and I-VDT-dispersion.

Figure 13 presents gaze data visualizations of an observer who watched triple_jump.avi in the Lund2013 dataset [15]. We utilized the gaze data of *triple_jump.avi* where the duration was 3 s. (a) is the visual stimulus. The black box is the moving target, and the red dots are raw gaze data. (b) to (f) show the visualizations after applying I-VT, I-DT, gaze behavior-based AlexNet, I-VDT, and REMoDNaV gaze-parsing algorithms, respectively. For REMoDNaV in (f), (a-1) and (a-2) were classified as smooth pursuits, and loss occurred since each smooth pursuit was depicted as one representative point. Therefore, (a-3) was not fully drawn in the heatmap, scanpath, and mid-level abstraction gaze data visualization. Except for REMoDNaV in (f), the mid-level abstraction gaze data visualizations in (b) to (e) revealed the distribution of missing data occurring in the heatmap and scanpath. Figure 14 presents the quantitative and qualitative comparisons of the parameter-based gaze-parsing algorithms used in Figure 13. Figure 14a presents the FQnS (Fixation Qualitative Score) of I-VT and I-DT. (b) shows the FQlS (Fixation Quantitative Score) of I-VT and I-DT. In (a) and (b), since I-DT utilized the dispersion and duration threshold as parameters, it was divided into I-DT-dispersion and I-DT-duration. FQnS and FQlS proposed by Komogortsev et al. [25] were employed to evaluate the fixation behavior detection performance. (c) and (d) show the PQnS (Smooth Pursuit Qualitative Score) of I-VDT. (c) presents PQnS_P, which calculated the score using the position difference of the smooth pursuit, and (d) gave PQnS_V, which measured the score using the velocity difference of the smooth pursuit. In (c) and (d), since I-VDT had the velocity and dispersion threshold as parameters, it was divided into I-VDT-velocity and I-VDT-dispersion. The PQnS proposed by Komogortsev and Karpov [64] compared the performance of smooth pursuit behavior detection using velocity and position as indicators. Range Coefficient (RC) was $RC=T*V_{i}+C$, where *T* is the threshold parameter value, $V_{i}$ is the increasing threshold value, and *C* is the initial threshold value. As seen in (a), FQnS had the maximum value when RC was 2 for I-VT and I-DT-dispersion and decreased after that. The I-DT-duration had the highest value at first and then continued to decrease. In (b), the I-DT-dispersion was not visible because it had FQlS values similar to that of I-VT. I-VT, I-DT-dispersion, and I-DT-duration had the maximum values when RC was 2 and decrease after that, which showed similar patterns as in (a). Additionally, the trends of the graphs were similar. Therefore, it was possible to obtain high performance of I-VT and I-DT identification classification by setting velocity, dispersion, and duration thresholds in the 2∼4 RC range as seen in (a) and (b). In (c) and (d), the graph patterns of I-VDT-dispersion and I-VDT-duration were similar. In both (c) and (d), the I-VDT-dispersion had high PQlS values when RC was between 14 and 30. The I-VDT-velocity had high PQlS values when RC was between 2 and 14. (c) and (d) show that the I-VDT performance was high when RC is 2∼14 for the velocity threshold, and RC was 14∼30 for the dispersion threshold. Machine learning-based algorithms were compared using the F1 score as presented in Table 2. The F1 score of gaze behavior-based AlexNet was 0.78 in the detection of *stare*, but unlike REMoDNaV, it did not detect eye movement events such as smooth pursuit. Likewise REMoDNaV does not detect stare. Therefore, it was not possible to compare the two algorithms directly.

## 6. Conclusions

In this paper, we compared the effects of gaze-parsing algorithms on gaze visualizations. Our abstract gaze movement visualization is an improved technique that emphasizes the directionality of eye movement in work proposed by Yoo et al. [6]. The visualizations utilized in this work include the heatmap, abstract gaze movement visualization, and scanpath visualization. The gaze-parsing algorithms we used in the comparison are velocity-based, dispersion-based, density-based, velocity and dispersion based, and parameter-free algorithms. The proposed gaze-parsing algorithms trains gaze behavior-based data using deep learning models for the classification. In this study, we chose AlexNet as the classification model by comparing the performance of CNN, AlexNet, and LeNet models, such as accuracy, loss, and training time. Eye movement events detection algorithms, such as I-VT, I-DT, DBSCAN with IQR, and I-VDT, vary significantly with different parameter settings. Additionally, although our proposed bahavior-based gaze data processing method sets parameters automatically, this gaze behavior parsing algorithm requires various case studies and evaluations to test the robustness.

Our proposed behavior-based gaze data processing method applies the AlexNet deep learning algorithm to train the gaze behavior image dataset. However, we did not consider the effect of sampling or window size on the deep learning model design. Since the gaze data sampling and the window size help to train the shape of the abstract gaze data, more studies on the sampling and window size might allow us to recommend a gaze-parsing algorithm according to the abstraction level of visualization. Additionally, we classified the data manually to create labeled training data. We divided the gaze behaviors into two classes, stare and move. Theoretically, the gaze data can be divided into more classes, such as noise and return, besides stare and move. In the manual labeling, however, we were not easily able to distinguish the sampled data. Even though the data were classified, the ratios of all classes were so different that it could not be trained. Therefore, we need various case studies and validation of this behavior-based gaze data processing method using AlexNet. Therefore, we plan to study the effect of data sampling and window size on the proposed gaze behavior-based data processing with AlexNet to recommend a gaze-parsing algorithm for each visualization abstraction level.

## Figures and Tables

**Table 1:** F1 scores are compared for the eye movement event identification between the Lund2013 dataset [15] with the 500-Hz sampling rate and the data collected in this work through Tobii Pro X2-30 with the 40-Hz sampling rate.

	Lund2013 Dataset (500 Hz)			Tobii Pro X2-30 Dataset (40 Hz)		
	Smooth Pursuit F1	Fixation F1	Saccade F1	Smooth Pursuit F1	Fixation F1	Saccade F1
I-VDT	0.42231	0.68683	0.52303	0.42803	0.58926	0.50061
REMoDNaV	0.52516	0.68745	0.52729	0.44115	0.68806	0.52281

**Table 2:** F1 scores of machine learning-based gaze-parsing algorithms.

	Fixation	Saccade	Smooth Pursuit	*Stare*	*Move*
Behavior-based	-	-	-	0.78080	0.542831
REMoDNaV	0.60656	0.43529	0.33945	-	-

## Data Availability

Not applicable.

## References

[1] Salvucci D.D., Goldberg J.H. Identifying fixations and saccades in eye-tracking protocols. Proceedings of the 2000 symposium on Eye Tracking Research & Applications.

[2] Olsen A. (2012). The Tobii I-VT Fixation Filter.

[3] Katsini C., Fidas C., Raptis G.E., Belk M., Samaras G., Avouris N. Eye gaze-driven prediction of cognitive differences during graphical password composition. Proceedings of the 23rd International Conference on Intelligent User Interfaces.

[4] de Urabain I.R.S., Johnson M.H., Smith T.J. (2015). GraFIX: A semiautomatic approach for parsing low-and high-quality eye-tracking data. Behav. Res. Methods.

[5] Yu M., Lin Y., Breugelmans J., Wang X., Wang Y., Gao G., Tang X. (2016). A spatial-temporal trajectory clustering algorithm for eye fixations identification. Intell. Data Anal..

[6] Yoo S., Jeong S., Kim S., Jang Y. (2019). Gaze Attention and Flow Visualization Using the Smudge Effect. Pacific Graphics (Short Papers).

[7] Ester M., Kriegel H.P., Sander J., Xu X. A Density-based Algorithm for Discovering Clusters a Density-based Algorithm for Discovering Clusters in Large Spatial Databases with Noise. Proceedings of the Second International Conference on Knowledge Discovery and Data Mining, KDD’96.

[8] Wooding D.S. Fixation maps: Quantifying eye-movement traces. Proceedings of the 2002 Symposium on Eye Tracking Research & Applications.

[9] Noton D., Stark L. (1971). Scanpaths in eye movements during pattern perception. Science.

[10] Holmqvist K., Nyström M., Andersson R., Dewhurst R., Jarodzka H., Van de Weijer J. (2011). Eye Tracking: A Comprehensive Guide to Methods and Measures.

[11] Kurzhals K., Fisher B., Burch M., Weiskopf D. Evaluating visual analytics with eye tracking. Proceedings of the Fifth Workshop on Beyond Time and Errors: Novel Evaluation Methods for Visualization.

[12] Blascheck T., Kurzhals K., Raschke M., Burch M., Weiskopf D., Ertl T. (2017). Visualization of Eye Tracking Data: A Taxonomy and Survey. Comput. Graph. Forum.

[13] Mital P.K., Smith T.J., Hill R.L., Henderson J.M. (2011). Clustering of gaze during dynamic scene viewing is predicted by motion. Cogn. Comput..

[14] Van Opstal A., Van Gisbergen J. (1987). Skewness of saccadic velocity profiles: A unifying parameter for normal and slow saccades. Vis. Res..

[15] Larsson L., Nyström M., Stridh M. (2013). Detection of saccades and postsaccadic oscillations in the presence of smooth pursuit. IEEE Trans. Biomed. Eng..

[16] Startsev M., Agtzidis I., Dorr M. (2019). 1D CNN with BLSTM for automated classification of fixations, saccades, and smooth pursuits. Behav. Res. Methods.

[17] Agtzidis I., Startsev M., Dorr M. Smooth pursuit detection based on multiple observers. Proceedings of the Ninth Biennial ACM Symposium on Eye Tracking Research & Applications.

[18] Larsson L., Nyström M., Andersson R., Stridh M. (2015). Detection of fixations and smooth pursuit movements in high-speed eye-tracking data. Biomed. Signal Process. Control..

[19] Ke S.R., Lam J., Pai D.K., Spering M. (2013). Directional asymmetries in human smooth pursuit eye movements. Investig. Ophthalmol. Vis. Sci..

[20] Robinson D.A., Gordon J., Gordon S. (1986). A model of the smooth pursuit eye movement system. Biol. Cybern..

[21] Bergstrom J.R., Schall A. (2014). Eye Tracking in User Experience Design.

[22] Stuart S., Hickey A., Vitorio R., Welman K., Foo S., Keen D., Godfrey A. (2019). Eye-tracker algorithms to detect saccades during static and dynamic tasks: A structured review. Physiol. Meas..

[23] Andersson R., Larsson L., Holmqvist K., Stridh M., Nyström M. (2017). One algorithm to rule them all? An evaluation and discussion of ten eye movement event-detection algorithms. Behav. Res. Methods.

[24] Komogortsev O.V., Jayarathna S., Koh D.H., Gowda S.M. Qualitative and quantitative scoring and evaluation of the eye movement classification algorithms. Proceedings of the 2010 Symposium on Eye-Tracking Research & Applications.

[25] Komogortsev O.V., Gobert D.V., Jayarathna S., Koh D.H., Gowda S.M. (2010). Standardization of automated analyses of oculomotor fixation and saccadic behaviors. IEEE Trans. Biomed. Eng..

[26] Schmidhuber J. (2015). Deep learning in neural networks: An overview. Neural Netw..

[27] Krizhevsky A., Sutskever I., Hinton G.E. (2012). Imagenet classification with deep convolutional neural networks. Adv. Neural Inf. Process. Syst..

[28] LeCun Y., Bottou L., Bengio Y., Haffner P. (1998). Gradient-based learning applied to document recognition. Proc. IEEE.

[29] Smith T.J., Mital P.K. (2013). Attentional synchrony and the influence of viewing task on gaze behavior in static and dynamic scenes. J. Vis..

[30] Löwe T., Stengel M., Förster E.C., Grogorick S., Magnor M. Visualization and analysis of head movement and gaze data for immersive video in head-mounted displays. Proceedings of the Workshop on Eye Tracking and Visualization (ETVIS).

[31] Wang X., Koch S., Holmqvist K., Alexa M. Tracking the gaze on objects in 3D: How do people really look at the bunny?. Proceedings of the SIGGRAPH Asia 2018 Technical Papers.

[32] Blignaut P., van Rensburg E.J., Oberholzer M. (2019). Visualization and quantification of eye tracking data for the evaluation of oculomotor function. Heliyon.

[33] Fujii K., Rekimoto J. SubMe: An Interactive Subtitle System with English Skill Estimation Using Eye Tracking. Proceedings of the 10th Augmented Human International Conference 2019.

[34] Otero-Millan J., Troncoso X.G., Macknik S.L., Serrano-Pedraza I., Martinez-Conde S. (2008). Saccades and microsaccades during visual fixation, exploration, and search: Foundations for a common saccadic generator. J. Vis..

[35] Martinez-Conde S., Otero-Millan J., Macknik S.L. (2013). The impact of microsaccades on vision: Towards a unified theory of saccadic function. Nat. Rev. Neurosci..

[36] Burch M., Andrienko G., Andrienko N., Höferlin M., Raschke M., Weiskopf D. Visual task solution strategies in tree diagrams. Proceedings of the 2013 IEEE Pacific Visualization Symposium (PacificVis).

[37] Eraslan S., Yesilada Y., Harper S. (2016). Eye tracking scanpath analysis techniques on web pages: A survey, evaluation and comparison. J. Eye Mov. Res..

[38] Peysakhovich V., Hurter C., Telea A. Attribute-driven edge bundling for general graphs with applications in trail analysis. Proceedings of the 2015 IEEE Pacific Visualization Symposium (PacificVis).

[39] Andrienko G., Andrienko N., Burch M., Weiskopf D. (2012). Visual analytics methodology for eye movement studies. IEEE Trans. Vis. Comput. Graph..

[40] Kurzhals K., Weiskopf D. Visualizing eye tracking data with gaze-guided slit-scans. Proceedings of the 2016 IEEE Second Workshop on Eye Tracking and Visualization (ETVIS).

[41] Peysakhovich V., Hurter C. (2018). Scanpath visualization and comparison using visual aggregation techniques. J. Eye Mov. Res..

[42] Ramdane-Cherif Z., NaÏt-AliNait-Ali A. (2008). An adaptive algorithm for eye-gaze-tracking-device calibration. IEEE Trans. Instrum. Meas..

[43] Wang K., Ji Q. Real time eye gaze tracking with 3d deformable eye-face model. Proceedings of the IEEE International Conference on Computer Vision.

[44] Hennessey C.A., Lawrence P.D. (2009). Improving the accuracy and reliability of remote system-calibration-free eye-gaze tracking. IEEE Trans. Biomed. Eng..

[45] Zhu Z., Ji Q. (2007). Novel eye gaze tracking techniques under natural head movement. IEEE Trans. Biomed. Eng..

[46] Button C., Dicks M., Haines R., Barker R., Davids K. (2011). Statistical modelling of gaze behaviour as categorical time series: What you should watch to save soccer penalties. Cogn. Process..

[47] Mazumdar D., Meethal N.S.K., George R., Pel J.J. (2021). Saccadic reaction time in mirror image sectors across horizontal meridian in eye movement perimetry. Sci. Rep..

[48] Krejtz K., Szmidt T., Duchowski A.T., Krejtz I. Entropy-based statistical analysis of eye movement transitions. Proceedings of the Symposium on Eye Tracking Research and Applications.

[49] Caldara R., Miellet S. (2011). i Map: A novel method for statistical fixation mapping of eye movement data. Behav. Res. Methods.

[50] Dink J.W., Ferguson B. (2015). eyetrackingR: An R Library for Eye-Tracking Data Analysis. www.eyetracking-r.com.

[51] Llanes-Jurado J., Marín-Morales J., Guixeres J., Alcañiz M. (2020). Development and calibration of an eye-tracking fixation identification algorithm for immersive virtual reality. Sensors.

[52] Liu W., Trapp A.C., Djamasbi S. (2021). Outlier-Aware, density-Based gaze fixation identification. Omega.

[53] Akshay S., Megha Y., Shetty C.B. Machine Learning Algorithm to Identify Eye Movement Metrics using Raw Eye Tracking Data. Proceedings of the 2020 Third International Conference on Smart Systems and Inventive Technology (ICSSIT).

[54] Zemblys R., Niehorster D.C., Holmqvist K. (2019). gazeNet: End-to-end eye-movement event detection with deep neural networks. Behav. Res. Methods.

[55] Blignaut P. (2009). Fixation identification: The optimum threshold for a dispersion algorithm. Atten. Percept. Psychophys..

[56] Urruty T., Lew S., Djeraba C., Simovici D.A. Detecting eye fixations by projection clustering. Proceedings of the 14th International Conference of Image Analysis and Processing-Workshops (ICIAPW 2007).

[57] Sugano Y., Matsushita Y., Sato Y. (2013). Graph-based joint clustering of fixations and visual entities. Acm Trans. Appl. Percept. (TAP).

[58] Soleh M.B., Anisa Y.H., Absor N.F., Edison R.E. Differences of Visual Attention to Memes: An Eye Tracking Study. Proceedings of the 1st Annual International Conference on Natural and Social Science Education (ICNSSE 2020).

[59] Srivastava N., Nawaz S., Newn J., Lodge J., Velloso E., Erfani S.M., Gasevic D., Bailey J. Are you with me? Measurement of Learners’ Video-Watching Attention with Eye Tracking. Proceedings of the LAK21: 11th International Learning Analytics and Knowledge Conference.

[60] Garro V., Sundstedt V. (2020). Pose and Visual Attention: Exploring the Effects of 3D Shape Near-Isometric Deformations on Gaze. J. WSCG.

[61] Jaeger L., Eckhardt A. (2021). Eyes wide open: The role of situational information security awareness for security-related behaviour. Inf. Syst. J..

[62] Nizam D.N.M., Law E.L.C. (2021). Derivation of young children’s interaction strategies with digital educational games from gaze sequences analysis. Int. J. Hum.-Comput. Stud..

[63] Tancredi S., Abdu R., Abrahamson D., Balasubramaniam R. (2021). Modeling nonlinear dynamics of fluency development in an embodied-design mathematics learning environment with Recurrence Quantification Analysis. Int. J. Child-Comput. Interact..

[64] Komogortsev O.V., Karpov A. (2013). Automated classification and scoring of smooth pursuit eye movements in the presence of fixations and saccades. Behav. Res. Methods.

[65] Dar A.H., Wagner A.S., Hanke M. (2020). REMoDNaV: Robust eye movement detection for natural viewing. BioRxiv.

